# Guy's Hospital

**Published:** 1905-07-22

**Authors:** 


### GUY'S HOSPITAL.

### THE OPENING OF THE GORDON MUSEUM.

At Guy's Hospital on Wednesday the New Gordon Museum of Pathological and Anatomical Subjects was opened. Proceedings commenced with a distribution of prizes to students by Dr. Pye-Smith, who congratulated the winners and gave a short but interesting address. He then, on behalf of the governors and medical staff, invited Mr. Gordon to accept a replica of the statue of Thomas Guy.

A vote of thanks was then proposed by Dr. Frederick Taylor, senior physician to the hospital, to Dr. Pye-Smith, which concluded the proceedings.

The hospital, residential college, Will's library, and new museum were then open for inspection. The Gordon Museum harbours a valuable collection where it can be displayed to proper advantage and to the immense benefit of medical education.

The building is arranged in four bays, lighted from the ceilings and intercommunicating; each bay contains wax models and specimens on tables and in wall-cases. Round each bay run two tiers of galleries, the colour scheme of the whole being in white and green.

The collection comprises specimens illustrating diseases of all the organs and systems of the body, in addition to which there is a case of various instruments and other objects of historical interest connected with Guy's Hospital.

The new museum was built from the plans of Mr. J. H. T. Wood, the hospital architect, and through its establishment, the specimens will no longer be scattered in different parts of the hospital.

